# Serum metabolomics study of the association between dairy intake and the anti-müllerian hormone annual decline rate

**DOI:** 10.1186/s12986-021-00591-y

**Published:** 2021-06-27

**Authors:** Nazanin Moslehi, Rezvan Marzbani, Hassan Rezadoost, Parvin Mirmiran, Fahimeh Ramezani Tehrani, Fereidoun Azizi

**Affiliations:** 1 Nutrition and Endocrine Research Center, Research Institute for Endocrine Sciences, Shahid Beheshti University of Medical Sciences, Tehran, Iran; 2 Department of Phytochemistry, Medicinal Plants and Drugs Research Institute, Shahid Beheshti University, Tehran, Iran; 3 Department of Clinical Nutrition and Dietetics, Faculty of Nutrition and Food Technology, National Nutrition and Food Technology Research Institute, Shahid Beheshti University of Medical Sciences, Tehran, Iran; 4 Reproductive Endocrinology Research Center, Research Institute for Endocrine Sciences, Shahid Beheshti University of Medical Sciences, Tehran, Iran; 5 Endocrine Research Center, Research Institute for Endocrine Sciences, Shahid Beheshti University of Medical Sciences, Tehran, Iran

**Keywords:** Anti-müllerian hormone, Branched-chain amino acids, Dairy, Mediation, Metabolomics, Ovarian reserve

## Abstract

**Background:**

Dairy intake has been implicated in later ovarian aging but mechanism underlying the association is unknown. This study aimed to investigate (1) associations between dairy intake and metabolites previously shown related to anti-müllerian hormone (AMH) decline rate; (2) mediating roles of these metabolites in the prospective association of total dairy consumption with odds of AMH fast decline rate.

**Methods:**

The participants comprised 186 reproductive-aged women randomly selected from the Tehran Lipid and Glucose Study. AMH was measured at baseline (1999–2001) and the 5th follow-up (2014–2017), and dietary data was collected at the second follow-up (2005–2008) using a food frequency questionnaire. Untargeted metabolomics was performed by gas chromatography–mass spectrometry using fasting-serum samples of the second follow-up. We analyzed dairy intake in association with the eight metabolites linked to the higher odds of AMH fast decline rate using linear regression with the Benjamini–Hochberg false discovery correction. Mediatory roles of the metabolites were assessed by bootstrapping.

**Results:**

Mean age and BMI of the participants at metabolomics assessment were 44.7 ± 5.87 years and 28.8 ± 4.88 kg/m^2^, respectively. Phosphate, branched-chain amino acids (BCAAs), and proline decreased significantly from the first to the third tertile of total dairy intake. Total dairy as a continuous variable inversely associated with phosphate (beta = −0.166; *p* value = 0.018), valine (beta = −0.176; *p* value = 0.016), leucine (beta = −0.226; *p* value = 0.002), proline (beta = −0.219; *p* value = 0.003), and urea (beta = −0.156; *p* = 0.035) after accounting for all potential covariates and correction for multiplicity (q-value < 0.1). Fermented dairy showed similar results, but milk did not associate with any of the metabolites. Simple mediation showed significant indirect effects for phosphate, proline, and BCAAs but not urea. Entering the sum of phosphate, proline, and BCAAs as a mediator, the metabolites' total indirect effects were significant [β = −0.12 (95% CIs − 0.26, − 0.04)]. In contrast, the direct association of total dairy intake with the fast decline in AMH was non-significant [β = −0.28 (95% CIs − 0.67, 0.10)].

**Conclusions:**

Total dairy was inversely associated with AMH decline rate-related metabolites. Inverse association of dairy intakes with the odds of AMH fast decline rate was indirectly mediated by lower phosphate, proline, and BCAAs.

## Background

During women's reproductive lifespan, fertility reduces with the gradual exhaustion of ovarian follicular pools till menopause occurs [1]. The reduction of this ovarian reserve is highly age-dependent, but the depletion rate varies among chronological age individuals [2–4]. Environmental factors, including nutrition, are thought to influence reproductive aging [5, 6]. A prospective study from the Nurses' Health Study (NHS) showed modestly later timing of menopause in women aged < 51 years with higher consumption of low-fat dairy and skim milk [7]. Higher intakes of low-fat dairy and skim milk in another prospective study from NHS ӀӀ, also related to the lower risk of early menopause [8]. Recently, we reported inverse prospective associations between total dairy, milk, and fermented dairy intakes and odds of anti-müllerian hormone (AMH) fast decline rate, as assessed by two-time point measurements of AMH during the study [9]. Since AMH is the best biomarker of ovarian reserve [10, 11], our findings reinforce the possible association of dairy intakes with ovarian aging.

Metabolomics is an emerging tool that can improve the understanding of the biological process, metabolic pathway related to nutrition, identifying biomarkers for diseases or specific exposures, including nutrition [12, 13]. We conducted a prospective study on associations of metabolites quantified by untargeted metabolomics approach using gas chromatography–mass spectrometry (GC–MS) with AMH decline rate to understand the metabolic pathway related to ovarian reserve reduction. The investigation suggests eight metabolites that are positively related to the odds of AMH fast decline rate [14]. In the present study, we aimed to assess the cross-sectional associations between dairy intakes and the eight metabolites related to the AMH fast decline rate. Furthermore, we aimed to explore whether the AMH decline rate-related metabolites mediate the association between dairy intake and AMH decline rate. Therefore, mediation analysis was used to evaluate the direct/indirect associations between total dairy intake and odds of AMH fast decline rate, considering the metabolites as mediators.

## Methods

### Participants

We conducted this research in the Tehran Lipid and Glucose Study (TLGS). The TLGS is a population-based cohort study in which 15,005 individuals aged ≥ 3 years recruited between 1999 and 2001 from residents of district 13 of Tehran, Iran's capital. Data on lifestyle factors and health of the participants have been reassessed every 3 years after the baseline survey [15, 16].

AMH concentrations of 1015 women aged 20–50 years who met eligibility criteria were measured at baseline. The eligibility criteria were having regular menstrual cycles and proven natural fertility, not having a history of hormonal disorders, i.e., polycystic ovarian syndrome, hysterectomy, or ovarian surgery. Among which 245 women were randomly selected to re-measure AMH concentrations at the 5th follow-up examination (2014–2017). As previously reported, the demographic and anthropometric characteristics of the 245 women, except for age that was higher, were similar to the other women who did not include in this study  [9]. Metabolomics analysis for 186 randomly selected women were done after excluding those with a history of hysterectomy or ovarian surgery (n = 7) during the follow-up and those without dietary data (n = 11). The random case selection function in SPSS was used to determine the participants in each step mentioned above. More details for the participants' selection were reported elsewhere [14]. The Ethics committee of the Research Institute for Endocrine Sciences at Shahid Beheshti University of Medical Sciences (IR.SBMU.ENDOCRINE.REC.A396.534) approved the study, and written informed consent was obtained from all the participants.

### AMH annual decline rate

Serum AMH concentrations were measured at the baseline and 5th follow-up examination by enzyme immunoassay (EIA) with Gen II kit (Beckman Coulter, Inc., CA, USA) and the Sunrise ELISA reader (Tecan Co. Salzburg, Austria). AMH decline rate was determined by subtracting the AMH values at the 5th follow-up examination from the baseline values, divided by the baseline values. This total AMH decline rate was divided by the duration of follow-up (in years) to estimate the AMH annual decline rate and expressed in percentages. The AMH fast decline rate was defined as yearly decline rates ≥ of 5.93% based on this variable's third tertile.

### Metabolomics analysis

Untargeted metabolomics analysis was conducted using the serum samples collected during the second follow-up examination (2005–2008). Sample preparation and derivatization, data acquisition, and data processing have been previously reported in detail. [14] In brief, iced methanol was added to 100 µl serum sample to extract metabolites and precipitate proteins. After drying the supernatant by blowing the pure nitrogen, oxiamtion with 50 µg methoxyamine hydrochloride in pyridine and then trimethylsilylation with 50 ml of *N*-methyl-*N*-trimethylsilyltrifluoroacetamide (MSTFA) with 1% trimethylchlorosilane were performed. The derivatized sample was injected in splitless mode into the DB-5MS column of gas chromatography to separate the metabolites. The single quadrupole mass spectrometer was used to detect mass using the electron impact (EI) ionization technique at 70 eV. Mass spectra were recorded at 0.4 scans/s with a range of *m*/*z* 45–450. Raw data were processed using the mass spectrometry-data independent analysis software (MS-DIAL, v.3.98). Metabolites were annotated using accurate mass and retention index (RI) with Mass Bank of North America (MoNA; http://mona.fiehnlab.ucdavis.edu), and manually checked by National Institute of Standards and Technology (NIST) library, the Golm Metabolome Database (GMD), and the Human Metabolome Database (HMDB). Peak height using the quantifier mass ion was used for quantification, which was normalized using succinic acid d-4 as an internal standard (peak height metabolite/peak height succinic acid d-4); the relative peak intensity was used in the statistical analyses. We previously found eight metabolites positively associated with odds of AMH fast decline rate, including *N*-Acetyl-d-glucosamine, phosphate, valine, leucine, isoleucine, proline, pyroglutamic acid, and urea [14]. In this study, we assessed the associations between the AMH decline rate-related metabolites and dairy intakes.

### Dietary assessment

Dietary data that were collected during the second follow-up (2005–2008) examination by the valid food frequency questionnaire (FFQ) was used in the current study. Daily dietary intakes of participants during the past year were estimated based on the frequency and amount of the 168 food items of the questionnaire. “low-fat milk”, “high-fat milk”, and “chocolate milk” were food items included for milk consumption. “Yoghurt”, “cheese”, “dough”, “kashk”, and “ice-cream” were food items included for estimated intakes of fermented dairy. Total dairy intakes were the sum of milk and fermented dairy consumptions. Intakes of these dairy groups were calculated as grams per energy intake of 1000 kcal (g/1000 kcal). A validity study suggested correlation coefficients of 0.59 in women for dairy products intakes assessed by the FFQ and multiple dietary recalls [17].

### Other variables

Demographic and reproductive information was gathered by interview using a predefined questionnaire. Anthropometric variables were measurements by trained staff at the TLGS unit, and body mass index (BMI) was calculated as weight (kg)/height (m^2^). Blood pressure was measured twice on the right arm with a mercury sphygmomanometer, and the mean of the two measures was used for the participants' blood pressure. Biochemical analyses were done using overnight fasting serum samples. Fasting blood glucose (FBG), triglycerides, total cholesterol, and high-density lipoprotein cholesterol (HDL-C) were measured by the enzymatic colorimetric method using Pars Azmon kits (Pars Azmon Inc., Tehran, Iran). In this study, data collected during the second follow-up examination (2005–2008) were used as covariates.

### Statistical analysis

Participants' characteristics at the second follow-up examination, when metabolomics analyses were done, were compared by the tertiles of total dairy intakes using analysis of variance (ANOVA) for normally distributed variables, the Kruskal–Wallis test for non-normally distributed variables, and chi-square for categorical variables.

Relative peak intensities of metabolites were transformed into the natural log (Ln) or square root and then autoscaled before statistical analyses. Relative peak intensities of metabolites across tertiles of dairy groups were compared using the general linear model (GLM), and results were reported as differences in least-square means considering the first tertile as a reference group. Association between metabolites (as continuous variables) and dairy groups were also examined using linear regression. Statistical analyses were performed using IBM SPSS Statistics (version 20; IBM Corp, Armonk, NY). The Benjamini–Hochberg procedure was applied to reduce the false discovery rate (FDR) due to multiplicity [18]. *p* value ≤ 0.05 and FDR-adjusted q-value ≤ 0.2 were defined as statistically significant.

Those metabolites were identified to be associated with dairy intake were selected. The mediating effects of the metabolites on the association between total dairy intakes and AMH fast decline rate were examined by PROCESS for SPSS macro (Version 3.5). The dependent variable was AMH fast decline (as a dichotomous variable), the independent was total dairy intakes, and mediators were the metabolites. The indirect effect was statistically tested with the bootstrap method based on 5000 simulations and 95% confidence intervals (CIs). The indirect effect of each metabolite was investigated separately; since the metabolites were highly correlated, we could not find their independent indirect (mediation) effects using multiple mediation analysis. Therefore, the sum of phosphate, proline, and BCAAs was calculated and used as the mediator to estimate the total indirect effects of the 5 metabolites. Standardized β (95% CIs) were reported for direct and indirect effects. In all statistical analyses, age, BMI, dietary intakes of energy, grains (g/1000 kcal), and legumes (g/1000 kcal) at the time of metabolomics analysis were accounted for as covariates.

## Results

### Participants’ characteristics

At the time of metabolomics analysis, the mean age and BMI of women were 44.7 ± 5.87 years and 28.8 ± 4.55 kg/m^2^, respectively, and 2.2% were smokers. Characteristics of the participants by tertiles of total dairy intake are shown in Table 1. No significant difference in age, BMI, waist circumference, and biochemical measurements were observed across the tertile of total dairy intake; the baseline value of AMH and the AMH annual decline rate were not significantly different across the tertiles. Furthermore, the frequency of individuals with diabetes did not significantly differ across tertiles of total dairy intake at metabolomics assessment. Mean intake of protein was higher, and grains were lower in women with higher intakes of total dairy intakes; the other dietary variables did not differ significantly.

**Table 1:** Characteristics of the participants by tertiles of total dairy intakes at metabolomics assessment^a^

Variables	Total dairy intakes (g/1000 kcal)			*p* value^b^
	Tertile 1 ≤ 146.4	Tertile 2 146.4–222.2	Tertile 3 > 222.2	
Number	62	61	63	
Age (years)	44.5 ± 5.30	44.0 ± 5.99	45.4 ± 6.28	0.397
Body mass index (kg/m^2^)	28.9 ± 4.78	28.7 ± 4.18	28.9 ± 4.73	0.967
Waist circumference (cm)	92.3 ± 10.9	91.3 ± 9.61	91.4 ± 10.6	0.839
Age at menarche (years)	13.4 ± 1.40	13.6 ± 1.35	13.3 ± 1.25	0.580
Baseline AMH (ng/ml)	0.72 (0.29, 2.04)	0.56 (4.56, 6.14)	0.58 (0.17, 1.54)	0.405
AMH annual decline rate (%/years)	5.81 (5.13, 6.10)	5.80(4.56, 6.14)	5.44 (4.83, 5.88)	0.066
Fasting blood sugar (mg/dl)	95.5 (86.8, 102)	92.0 (86.0, 96.5)	92.0 (87.0, 98.0)	0.099
Triglycerides (mg/dl)	117 (82.3, 151)	113 (79.0, 144)	125 (71.0, 173)	0.825
Total cholesterol (mg/dl)	189 ± 32.7	189 ± 35.5	197 ± 34.5	0.366
HDL-C (mg/dl)	48.4 ± 11.2	49.8 ± 11.1	49.7 ± 10.8	0.724
LDL-C (mg/dl)	114 ± 30.0	115 ± 32.8	119 ± 29.3	0.635
Systolic blood pressure (mmHg)	110 ± 11.4	110 ± 17.3	114 ± 18.9	0.334
Diastolic blood pressure (mmHg)	74.1 ± 9.41	75.1 ± 9.57	76.4 ± 10.3	0.413
Diabetes^c^	7 (11.5)	4 (7.1)	3 (5.1)	0.418
*Daily dietary intakes*				
Energy (kcal)	2342 ± 814	2269 ± 755	2217 ± 855	0.686
Carbohydrate (% of energy)	58.8 ± 7.14	57.2 ± 6.20	55.9 ± 7.53	0.066
Protein (% of energy)	12.4 ± 2.33	13.2 ± 1.77	14.8 ± 2.11	< 0.001
Fat (% of energy)	31.5 ± 7.46	31.9 ± 6.67	32.9 ± 6.33	0.526
Grains (g/1000 kcal)	195 ± 53.4	169 ± 61.7	154 ± 55.3	< 0.001
Legumes (g/1000 kcal)	13.3 ± 10.9	17.6 ± 18.9	12.5 ± 11.5	0.103
Meats (g/1000 kcal)	24.1 ± 17.4	20.4 ± 10.6	26.7 ± 16.1	0.071
Fruits (g/1000 kcal)	169 ± 137	158 ± 108	174 ± 86.7	0.726
Vegetables (g/1000 kcal)	145 ± 60.6	179 ± 136	169 ± 75.8	0.065

*AMH* anti-Müllerian hormone, *HDL-C* high-density lipoprotein cholesterol, *LDL-C* low-density lipoprotein cholesterol

^a^Data are presented as mean ± SD, median (quartile 1, quartile 3), and number (%)

^b^Based on analysis of variance for normally-distributed variables and Kruskal–Wallis test for non-nomally distributed variables

^c^Diabetes status was determined for n = 176 based on the American Diabetes Association criteria

### Associations between dairy groups and AMH decline rate-related metabolites

Least-squares means of 8 metabolites related to AMH fast decline rate across tertile categories of dairy groups are shown in Table 2. After adjusting for potential covariates including age, BMI, energy intake, grains and legumes intakes, the relative abundance of phosphate, valine, leucine, isoleucine, and proline was significantly lower with higher intakes of total dairy intakes. The findings were similar for fermented dairy intakes except for isoleucine that was non-significant (p-trend = 0.09); the associations remained significant after correction for multiple testing. However, the concentrations of the metabolites did not differ significantly across the tertiles of milk intakes.

**Table 2:** Relative abundance of the 8 metabolites related to the AMH decline rate by tertiles of dairy intake^a^

Dairy groups	Metabolites^b^	Tertile 1	Tertile 2	Tertile 3	p-trend	q-value^c^
Total dairy	*N*-Acetyl-d-glucosamine	Ref	− 0.340 (− 0.678, − 0.003)	− 0.313 (− 0.656, 0.030)	0.068	0.154
	Phosphate	Ref	− 0.435 (− 0.770, − 0.100)	− 0.433 (− 0.774, − 0.093)	0.01	0.048
	Valine	Ref	− 0.467 (− 0.818, − 0.116)	− 0.492 (− 0.848, − 0.135)	0.006	0.048
	Leucine	Ref	− 0.523 (− 0.867, − 0.180)	− 0.636 (− 0.986, − 0.287)	< 0.001	0.012
	Isoleucine	Ref	− 0.384 (− 0.732, − 0.037)	− 0.465 (− 0.818, − 0.112)	0.009	0.048
	Proline	Ref	− 0.503 (− 0.850, − 0.155)	− 0.657 (− 1.01, − 0.304)	< 0.001	0.012
	Pyroglutamic acid	Ref	− 0.289 (− 0.647, 0.069)	− 0.229 (− 0.593, 0.135)	0.203	0.287
	Urea	Ref	− 0.323 (− 0.680, 0.035)	− 0.319 (− 0.683, 0.044)	0.079	0.154
Fermented dairy	*N*-Acetyl-d-glucosamine	Ref	− 0.102 (− 0.442, 0.237)	− 0.212 (− 0.554, 0.130)	0.223	0.289
	Phosphate	Ref	− 0.166 (− 0.503, 0.171)	− 0.382 (− 0.721, − 0.042)	0.027	0.081
	Valine	Ref	− 0.087 (− 0.441, 0.266)	− 0.408 (− 0.764, − 0.502)	0.022	0.081
	Leucine	Ref	− 0.031 (− 0.382, 0.320)	− 0.388 (− 0.741, − 0.034)	0.026	0.081
	Isoleucine	Ref	− 0.027 (− 0.378, 0.324)	− 0.295 (− 0.648, 0.058)	0.090	0.154
	Proline	Ref	0.012 (− 0.343, 0.367)	− 0.374 (− 0.731, − 0.016)	0.032	0.085
	Pyroglutamic acid	Ref	− 0.041 (− 0.400, 0.318)	− 0.217 (− 0.489, 0.235)	0.483	0.504
	Urea	Ref	− 0.013 (− 0.370, 0.345)	− 0.307 (− 0.666, 0.053)	0.082	0.154
Milk	*N*-Acetyl-d-glucosamine	Ref	0.128 (− 0.212, 0.468)	− 0.195 (− 0.536, 0.147)	0.188	0.282
	Phosphate	Ref	0.110 (− 0.231, 0.450)	− 0.220 (− 0.562, 0.122)	0.146	0.233
	Valine	Ref	0.108 (− 0.253, 0.469)	− 0.033 (− 0.395, 0.330)	0.774	0.774
	Leucine	Ref	0.126 (− 0.230, 0.483)	− 0.176 (− 0.534, 0.182)	0.250	0.300
	Isoleucine	Ref	0.111 (− 0.244, 0.466)	− 0.110 (− 0.467, 0.246)	0.451	0.504
	Proline	Ref	0.107 (− 0.254, 0.467)	− 0.190 (− 0.552, 0.172)	0.229	0.289
	Pyroglutamic acid	Ref	− 0.099 (− 0.458, 0.261)	− 0.310 (− 0.671, 0.051)	0.084	0.154
	Urea	Ref	0.044 (− 0.319, 0.406)	− 0.115 (− 0.479, 0.249)	0.480	0.504

^a^Data is reported as differences in least-squares means of relative abundance of metabolites compared to the tertile 1 as the reference group after adjustment for age, BMI, energy intake, grains and legumes intakes

^b^Metabolites values are natural log or square root-transformed and auto-scaled

^c^*p* value corrected for multiple comparisons using Benjamini–Hochberg procedure

Associations between dairy groups and AMH decline rate–related metabolites are shown in Table 3. All metabolites were inversely associated with total dairy intakes in unadjusted models. The associations remained significant after accounting for age and BMI of the participants and correction for multiplicity (FDR-adjusted q-value < 0.2). However, after entering other dietary variables as covariates, the associations remained significant for phosphate, valine, leucine, proline, and urea. Higher intakes of fermented dairy were associated with a lower abundance of valine, leucine, proline, and urea after adjusting for all potential covariates (model 2). In the age and BMI-adjusted model, milk intake was inversely associated with pyroglutamic acid. However, the association became non-significant after controlling for other dietary variables.

**Table 3:** Standardized β for associations between dairy intakes and AMH decline rate-related metabolites^a,b^

Metabolites (HMDB ID)	Total diary			Fermented dairy			Milk		
	β	*p* value	q-value	β	*p* value	q-value	β	*p* value	q-value
*N-Acetyl-d-glucosamine (HMDB0000215)*									
Unadjusted	− 0.160	0.029	0.05	− 0.111	0.132	0.168	− 0.107	0.145	0.174
Model 1	− 0.156	0.023	0.046	− 0.095	0.169	0.193	− 0.118	0.085	0.113
Model 2	− 0.107	0.129	0.194	− 0.051	0.461	0.475	0.091	0.192	0.243
*Phosphate (HMDB0001429)*									
Unadjusted	− 0.217	0.003	0.010	− 0.186	0.011	0.024	− 0.111	0.131	0.168
Model 1	− 0.213	0.002	0.008	− 0.171	0.014	0.031	− 0.122	0.082	0.113
Model 2	− 0.166	0.018	0.062	0.131	0.061	0.133	− 0.093	0.187	0.243
*Valine* (HMDB0000883)									
Unadjusted	− 0.215	0.003	0.010	− 0.236	0.001	0.005	− 0.061	0.409	0.427
Model 1	− 0.207	0.004	0.014	− 0.215	0.001	0.008	− 0.073	0.312	0.326
Model 2	− 0.176	0.016	0.062	− 0.173	0.017	0.062	− 0.064	0.380	0.456
*Leucine* (HMDB0000687)									
Unadjusted	− 0.259	< 0.001	0.005	− 0.237	0.001	0.005	− 0.119	0.106	0.159
Model 1	− 0.254	< 0.001	0.008	− 0.221	0.002	0.008	− 0.129	0.072	0.108
Model 2	− 0.226	0.002	0.036	− 0.186	0.010	0.060	− 0.118	0.108	0.185
*Isoleucine* (HMDB0000172)									
Unadjusted	− 0.163	0.026	0.048	− 0.173	0.018	0.036	− 0.051	0.485	0.485
Model 1	− 0.156	0.027	0.050	− 0.153	0.032	0.055	− 0.064	0.372	0.372
Model 2	− 0.128	0.078	0.144	− 0.111	0.122	0.194	− 0.061	0.399	0.456
*Proline* (HMDB0000162)									
Unadjusted	− 0.245	0.001	0.005	− 0.245	0.001	0.005	− 0.092	0.211	0.241
Model 1	− 0.240	0.001	0.008	− 0.230	0.002	0.008	− 0.101	0.166	0.193
Model 2	− 0.219	0.003	0.036	− 0.189	0.010	0.060	− 0.107	0.147	0.208
*Pyroglutamic acid* (HMDB0000267)									
Unadjusted	− 0.191	0.009	0.021	− 0.117	0.113	0.160	− 0.143	0.052	0.083
Model 1	− 0.186	0.010	0.024	− 0.102	0.161	0.193	− 0.152	0.035	0.056
Model 2	− 0.137	0.061	0.133	− 0.052	0.475	0.475	− 0.131	0.073	0.144
*Urea* (HMDB0000294)									
Unadjusted	− 0.203	0.006	0.016	− 0.207	0.005	0.015	− 0.071	0.333	0.363
Model 1	− 0.199	0.006	0.018	− 0.198	0.007	0.019	− 0.078	0.287	0.303
Model 2	− 0.156	0.035	0.093	− 0.154	0.035	0.093	− 0.057	0.440	0.475

Model 1 adjusted for age and BMI. Model 2 adjusted for age, BMI, energy intake, grains and legumes intake. *AMH* anti-Müllerian hormone, *HMDB* Human Metabolome Database

^a^Metabolites values were natural log or square root-transformed and auto-scaled

^b^The metabolites were positively related to the AMH fast decline rate

### Mediating analysis

The mediating effects of phosphate, BCAAs, proline, and urea, the metabolites associated with total dairy intakes based on linear regression, on the association between total dairy intakes and AMH fast decline rate are reported in Table 4. After adjusting for age, BMI, energy, grains, and legumes intakes, the indirect effects of phosphate, proline, and BCAAs were significant. The direct effects of total dairy intakes were not significant in any of the simple mediation models. The sum of phosphate, proline, and BCAAs was calculated and used as a mediator to estimate the total indirect effects of the 5 metabolites. Total dairy intake was inversely associated with the pooled estimation of the five metabolites [β = −0.93 (95% CIs −1.58, − 0.28; *p* = 0.005)], and the metabolites were positively related to fast decline in AMH [β = 0.13 (95% CIs 0.05, 0.22; *p* = 0.002)] after adjusting for covariates. The metabolites' total indirect effects were significant, while the direct effect of total dairy intake on the fast decline in AMH was non-significant [β = −0.28 (95% CIs − 0.67, 0.10); *p* = 0.152].

**Table 4:** Mediation effects of dairy intakes on AMH fast decline rate through metabolites^a,b^

Metabolite^c^	Effect of M on Y^d^		Indirect effect^f^	
	Βeta^e^ (95% CIs)	*p* value	Beta (95% CIs)	SE
Phosphate	0.66 (0.26, 1.06)	0.001	− 0.11 (− 0.24, − 0.03)	0.059
BCAAs	0.20 (0.07, 0.33)	0.003	− 0.11 (− 0.25, − 0.02)	0.053
Proline	0.40 (0.07, 0.74)	0.019	− 0.09 (− 0.22, − 0.01)	0.051
Urea	0.31 (− 0.02, 0.65)	0.064	− 0.05 (− 0.15, 0.003)	0.042
Phosphate + BCAAs + proline	0.13 (0.05, 0.22)	0.002	− 0.12 (− 0.26, − 0.04)	0.057

*AMH* anti-Müllerian hormone, *BCAAs* Branched-chain amino acids

^a^Adjusted for age and BMI at metabolomics measurement, energy intake, dietary intakes of legumes and grains (g/1000 kcal)

^b^The AMH fast decline rate was defined as yearly decline rates ≥ of 5.93% based on this variable's third tertile

^c^Natural log or square root-transformed

^d^Effects of mediators (metabolites) on AMH fast decline rate

^e^Standardized value

^f^Estimated using boot-strapping

## Discussion

In this study, we examined cross-sectional associations between dairy products and metabolites previously found to be related to the higher odds of AMH fast decline rate. After adjusting for age, BMI, intakes of energy, grains, and legumes the intensities of five AMH decline rate-related metabolites including phosphate, valine, leucine, isoleucine, and proline reduced significantly from the first to the third tertile of total dairy intake. Total dairy intakes as a continuous variable were also inversely associated with phosphate, valine, leucine, proline, and urea. Using mediating analyses, we identified that phosphate, BCAAs, and proline fully mediated the prospective inverse association of total dairy intakes and odds of AMH fast decline rate.

Reduced fertility, end of fertility, menstrual cycle irregularity, and menopause are events that women experience during their reproductive life span due to the gradual loss of numbers of ovarian follicles with aging [1]. Though the ovarian aging process naturally occurs in all women, the rates of follicular loss and the age at which the reproductive events happen considerably vary [1–4]. As fast ovarian reserve decline rate and early menopause increases the risk of cardiovascular diseases, diabetes, neurological diseases, and mortality [19–21], understanding modifiable determinants of ovarian aging are clinically important for managing fertility and other health risks in women. Few studies investigated the association between dairy intake and timing of menopause [7, 8, 22, 23]; among them, two studies showed the later timing of menopause with higher intakes of low-fat dairy and skimmed milk [7, 8]. We previously examined the prospective association between dairy intake and the annual decline rate of AMH as the best biomarkers of ovarian reserve. Findings of the study suggested inverse associations of total dairy, fermented dairy, and milk intakes and odds of AMH fast decline rate [9]. To study molecular biology underlying the association, we evaluated the associations between dairy intake and AMH decline rate-related metabolites in the current study.

Finding objective markers to assess dairy consumption accurately is of interest to better understand this heterogeneous food group's health effects. Some metabolomics studies have been conducted to find biomarkers reflecting habitual dairy consumption in free-living individuals [24–27]. These studies have proposed numerous metabolites, the majority of which were lipids, related to total dairy or specific dairy foods intakes. However, no reliable and validated biomarkers for dairy intake have been identified to date [28]. It is unclear that the dairy-related metabolites are indicative of dietary intakes or dairy-induced changes in metabolism. Our findings suggest that higher dairy intake are associated with lower circulatory phosphate, BCAAs, and proline. These findings reflect the metabolic pathways through which dairy may associate with AMH decline rate which is beyond markers of its dietary intake.

Approximately 21% of the amino acid residue of dairy protein is BCAAs, and therefore the food group is an important dietary source of the essential amino acids [29]. Proline, a non-essential amino-acids, is also the second most abundant amino acid in a dairy protein with a proportion of 9%. The inverse association between dairy and serum BCAAs and proline was observed in the study, unlike that the food groups are one of the rich sources of these amino-acids. However, it is unclear whether dietary intakes of the amino-acids influence their fasting circulatory levels as the number of the studies is limited, and their findings are inconsistent [30–33]. A prospective study showed higher plasma levels of BCAAs with their higher dietary intakes [30]. A cross-sectional study also reported positive associations for total animal protein and red meat with plasma BCAAs, but dairy protein intake was unrelated to the blood levels of BCAAs [33]. No significant association was observed between dietary and fasting plasma levels of BCAAs in another cross-sectional study [31]. Short term changes in dairy intakes by 1.5–2 serving of daily milk intake for one month could not affect plasma BCAAs, proline, and other free amino acids in an intervention study of 102 healthy adults [32]. Whereas the BCAAs catabolism is tightly regulated, increased dietary intakes of BCAAs have been proposed not to contribute a remarkable elevation in their circulatory levels in prandial state [34, 35]. In addition to dietary intake, protein turnover and gene expression of activity of the branched-chain-keto acid dehydrogenase (BCKAD), the rate-limiting enzyme of BCAAs catabolism, also influence their circulatory levels [34, 36]. Although the cause and effect relationship between insulin resistance and elevated BCAA is unknown, it is believed that insulin activates BCKD, so insulin resistance leads to the decreased catabolism of the amino-acids [35]. Associations of dairy intakes with gene expression and activity of the enzyme have not been studied, but dairy intakes through improved insulin sensitivity [37] can be related to the lower circulatory BCAAs.

Type of milk, the microorganism, and process increase diversity of dairy products. The health effects of the diverse food groups may be different [38]. In this study, we categorized dairy products into two groups of fermented dairy and milk. Considering the subtypes, the inverse association between milk intake and AMH decline rate metabolites were not significant. In our participants, fermented dairy is a dominant type of dairy food consumed, and milk constitutes only 36.2% of total dairy intakes. This amount of milk consumption may be inadequate to find any significant relationship with the metabolites. Since the milk and fermented dairy could reduce the odds of AMH fast decline rate to almost the same extent [9], the fermentation process might not promote these dairy-metabolite associations.

In our study, total dairy intake could explain the variance of AMH decline rate-related metabolites modestly, ranging from 2.6% in phosphate to 6.7% in leucine, so dairy intake might not be the main variable influencing these metabolites. However, the mediating analyses showed that phosphate, proline, and BCAAs mediated the prospective association between total dairy intake and odds of AMH fast decline rate. In other words, higher dairy intake through the lower abundance of phosphate, proline, and BCAAs was associated with lower odds of AMH fast decline.

Our findings provided some biological insights supporting the hypothesis that dairy intake may reduce the AMH decline rate. However, the limitations of the study should be considered. First, habitual dairy intake was indicated using FFQ, which is prone to recall bias. Second, metabolomics analysis was performed using serum samples collected at 2005–2008 and stored at − 80. Though the stability of metabolites at this temperature is different, in general, the prolonged storage of samples has been shown to change the concentrations of the metabolites, which can be a potential source of bias in a data set [39, 40]. Third, we cannot conclude any causal effects of dairy intakes on AMH decline rates-related metabolites based on the cross-sectional analyses. Fourth, the metabolites were highly correlated, and therefore we could not examine their independent mediating roles using multiple mediation analysis. Fifth the annual AMH decline rate was calculated based on AMH values measured at the baseline and the fifth follow-up and assuming a fixed pattern for decline rate; while an overtime intra-individual variations may be proposed.

## Conclusions

In conclusion, after accounting for age, BMI, and dietary intakes, higher total dairy consumption was associated with lower intensities of serum phosphate, proline, and BCAAs, and urea, metabolites that positively related to the AMH decline rate. Lower concentrations of phosphate, proline, and BCAAs indirectly mediated the prospective inverse association of dairy intakes and odds of AMH fast decline rate. The study's findings provide some knowledge on the metabolites that may be involved in the association between dairy intake and ovarian aging, which can be confirmed using a targeted metabolomics approach. Further investigation in other populations using a combination of metabolomics platforms increases the numbers of metabolites related to the dairy-ovarian aging association.

## Data Availability

The dataset analyzed during the current study are available from the corresponding author on upon request.

## Abbreviations

ANOVA: Analysis of variance
AMH: Anti-Müllerian hormone
BCAAs: Branched-chain amino acids
EI: Electron impact
EIA: Enzyme immunoassay
FBG: Fasting blood glucose
FDR: False discovery rate
FFQ: Food frequency questionnaire
GC–MS: Gas chromatography–mass spectrometry
GLM: General linear model
GMD: Golm Metabolome Database
HDL-C: High-density lipoprotein cholesterol
HMDB: Human Metabolome Database
NIST: National Institute of Standards and Technology
NHS: Nurses' Health Study
MoNA: Mass Bank of North America
MSTFA: *N*-Methyl-*N*-trimethylsilyltrifluoroacetamide
TLGS: Tehran Lipid and Glucose Study
